# White matter damage, neuroinflammation, and neuronal integrity in HAND

**DOI:** 10.1007/s13365-018-0682-9

**Published:** 2018-10-05

**Authors:** Aljoharah Alakkas, Ronald J. Ellis, Caitlin Wei-Ming Watson, Anya Umlauf, Robert K. Heaton, Scott Letendre, Ann Collier, Christina Marra, David B. Clifford, Benjamin Gelman, Ned Sacktor, Susan Morgello, David Simpson, J. Allen McCutchan, Asha Kallianpur, Sara Gianella, Thomas Marcotte, Igor Grant, Christine Fennema-Notestine, for the CHARTER Group

**Affiliations:** 1 University of California at San Diego, La Jolla, CA USA; 2 University of Washington, Seattle, WA USA; 3 Washington University School of Medicine, St. Louis, MO USA; 4 University of Texas Medical Branch, Galveston, TX USA; 5 The Johns Hopkins University School of Medicine, Baltimore, MD USA; 6 The Mount Sinai Hospital, New York, NY USA; 7 Cleveland Clinic and Lerner Research Institute, Cleveland, OH USA

**Keywords:** HAND, MRI

## Abstract

HIV-associated neurocognitive disorders (HANDs) persist even with virologic suppression on combination antiretroviral therapy (cART), and the underlying pathophysiological mechanisms are not well understood. We performed structural magnetic resonance imaging and MR spectroscopy (MRS) in HIV+ individuals without major neurocognitive comorbidities. Study participants were classified as neurocognitively unimpaired (NU), asymptomatic (ANI), mild neurocognitive disorder (MND), or HIV­associated dementia (HAD). Using structural MRI, we measured volumes of cortical and subcortical gray matter and total and abnormal white matter (aWM). Using single-voxel MRS, we estimated metabolites in frontal gray matter (FGM) and frontal white matter (FWM) and basal ganglia (BG) regions. Adjusted odds ratios were used to compare HAND to NU. Among 253 participants, 40% met HAND criteria (21% ANI, 15% MND, and 4% HAD). Higher risk of HAND was associated with more aWM. Both HAD and MND also had smaller gray and white matter volumes than NU. Among individuals with undetectable plasma HIV RNA, structural volumetric findings were similar to the overall sample. MND had lower FWM creatine and higher FGM choline relative to NU, whereas HAD and ANI had lower BG *N*­acetyl aspartate relative to NU. In the virologically suppressed subgroup, however, ANI and MND had higher FGM choline compared to NU. Overall, HAND showed specific alterations (more aWM and inflammation; less gray matter volume and lower NAA). Some MR measures differentiated less severe subtypes of HAND from HAD. These MR alterations may represent legacy effects or accumulating changes, possibly related to medical comorbidities, antiretroviral therapy, or chronic effects of HIV brain infection.

## Introduction

Combination antiretroviral treatment (cART) has markedly increased longevity among individuals infected with the human immunodeficiency virus (HIV) (Justice 2010; Luther and Wilkin 2007). As the HIV+ population ages, understanding HIV-associated chronic diseases becomes increasingly relevant. Despite successful virologic suppression with cART, cognitive deficits referred to as HIV-associated neurocognitive disorders (HANDs) persist, affecting up to 50% of those living with HIV (Robertson et al. 2007; Sacktor et al. 2016; Heaton et al. 2010). Similarly, neuroimaging abnormalities such as more white matter disease (e.g., T2 hyperintensities on structural magnetic resonance imaging) and lower neuronal integrity (e.g., *N*-acetyl-aspartate on MR spectroscopy (MRS)) persist in many individuals. While numerous prior studies describe the relationship of these abnormalities to HAND generally (Masters and Ances 2014; Holt et al. 2012), few have evaluated MR measures based on the HAND classification scheme (Rahimian and He 2017).

The Frascati research criteria (Antinori et al. 2007) distinguish three HAND subtypes by the severity of neurocognitive impairment (NCI) and the presence and severity of disability in activities of daily living. While the prevalence of the most severe form, HIV-associated dementia (HAD), has declined substantially with cART (< 5%), the milder forms of HAND persist (Heaton et al. 2010). The most common form is asymptomatic neurocognitive impairment (ANI, 70%); individuals with ANI perform in the impaired range in at least two cognitive domains on neuropsychological testing, but do not report functional impairment (Heaton et al. 2010; Antinori et al. 2007). Those with mild neurocognitive disorder (MND, 25%) have cognitive deficits and experience mild to moderate impairment in everyday functioning (Heaton et al. 2010; Antinori et al. 2007). Both ANI and MND show poorer functioning in financial (Heaton et al. 2004) and medication management (Hinkin et al. 2002), greater unemployment (Woods et al. 2011), and early mortality (Ellis et al. 1997).

This study’s primary aim was to identify imaging alterations that relate to HAND subtypes relative to cognitively unimpaired HIV+ individuals. We evaluated structural and metabolite neuroimaging biomarkers, and we separately evaluated a subset with virologic suppression on cART. We hypothesized that cognitively impaired HIV+ individuals would have less cortical and subcortical gray matter, less total white matter, and more abnormal white matter (aWM) compared to cognitively unimpaired HIV+ individuals. In mid-frontal cortex (FGM), frontal white matter (FWM), and basal ganglia (BG), we evaluated single-voxel MRS metabolites reflecting neuronal integrity and inflammation, including the following: *N*-acetyl aspartate (NAA), a marker of neuronal viability and integrity; choline (Cho), a marker of cell membrane degradation and inflammation; creatine (Cr), a marker of energy metabolism; and myoinositol (MI), a marker of glial proliferation and gliosis. We hypothesized that cognitively impaired HIV+ individuals would have lower NAA, higher Cho, and higher MI in FGM, FWM, and BG relative to cognitively unimpaired HIV+ individuals. We predicted that brain structural and metabolic findings would differ between neurocognitively unimpaired (NU), ANI, MND, and HAD groups, with the largest differences between HAD and the other cognitive groups. We evaluated these hypotheses in a large cohort of prospectively studied HIV+ individuals, most on cART, with neurocognitive, clinical, structural MRI, and MRS methods.

## Methods

### Overview of study design

The CNS HIV Antiretroviral Therapy Effects Research (CHARTER) study is a multicenter longitudinal study that recruited HIV+ participants at varying stages of the disease (Heaton et al. 2010). A subset of CHARTER participants underwent brain imaging and had longitudinal follow-up with semi-annual evaluations. The current study used baseline brain imaging data as in (Jernigan et al. 2011).

### Study population

The 253 participants were studied from May 2004 to August 2007 at five participating sites: Johns Hopkins University (Baltimore, MD, *n* = 38), Mt. Sinai School of Medicine (New York, NY, *n* = 57), University of California at San Diego (San Diego, CA, *n* = 73), University of Texas Medical Branch (Galveston, TX, *n* = 56), and University of Washington (Seattle, WA, *n* = 29). The sample was a subset of the HIV+ CHARTER cohort who agreed to undergo neuroimaging (Heaton et al. 2010; Jernigan et al. 2011). Other aspects of the neuroimaging findings in this cohort have been previously published (Archibald et al. 2014). As described in our original baseline neuroimaging study of HIV infection (Woods et al. 2011), we excluded participants with gross morphological abnormalities not consistent with HIV-related neuropathology, comorbid conditions that could fully explain cognitive impairment (Heaton et al. 2010; Woods et al. 2011), a history of AIDS-defining opportunistic infection of the central nervous system (CNS), CNS malignancies, or severe psychiatric conditions that might preclude adequate completion of the study assessments (Heaton et al. 2010; Woods et al. 2011). The Human Subjects Protection Committees of each participating institution approved all procedures, and written informed consent was obtained from all study participants.

### Study procedures

#### Neuromedical assessment

The assessment included a medical history, neurological physical examination, and laboratory assessment. Medical history included demographics and current and past exposure to specific cART drugs. Blood was collected via phlebotomy and cerebrospinal fluid (CSF) via lumbar puncture. Laboratory assessment included measurement of complete blood counts, rapid plasma reagin (RPR), blood and CSF HIV RNA via RT-PCR ultrasensitive assay, current CD4 measured by flow cytometry, and hepatitis C virus antibody. Nadir CD4+ T cell count was based on a combination of self-report and medical records. CSF escape was defined as CSF HIV RNA > 50 copies/mL when plasma HIV RNA was < 50 copies/mL, and CSF discordance as HIV RNA in CSF at least 0.5 log_10_ copies greater than in plasma.

#### Neurobehavioral assessment

All participants underwent neuropsychological testing and completed functional status reports; previous work has described the CHARTER neurobehavioral assessment in detail (Heaton et al. 2010). The neuropsychological tests include assessment of seven putative cognitive domains sensitive to the detection of HIV-associated CNS dysfunction. Functional status was assessed using the Patient’s Assessment of Own Functioning Inventory (PAOFI), a modified version of the Lawton and Brody Scale, an employment questionnaire, and performance-based measures. To classify severity of neurocognitive impairment, Frascati criteria for diagnosing impairment were used (Antinori et al. 2007; Clifford and Ances 2013). The Frascati criteria for global neurocognitive impairment require impairment in at least two of the seven cognitive domains. All impaired individuals were classified based on their degree of cognitive impairment and functional impairment (Heaton et al. 2010).

#### Multi-channel structural MRI

The following volumes were measured by structural neuroimaging: total cerebral white matter and abnormal white matter, cerebral cortical and subcortical gray smatter, and supratentorial cranial vault. The details related to image sequences and analyses for multi-channel structural MRI were described previously (Jernigan et al. 2011; Fennema-Notestine et al. 2013); we provide an overview here. All imaging was performed on General Electric (GE) 1.5-T scanners that were annually reviewed for quality. Scanner was included as a covariate in the statistical modeling as in previous CHARTER studies (Jernigan et al. 2011; Fennema-Notestine et al. 2013). Four series were acquired including two coronal acquisitions with a thickness of 2 mm and two sagittal acquisitions with a thickness of 1.5 mm. The coronal sequences were 2D T2-weighted and the proton density (PD)-weighted fast spin echoes (FSEs). The sagittal sequences included a T1-weighted and a PD-weighted spoiled gradient recalled acquisition (SPGR).

As described previously (Jernigan et al. 2011; Fennema-Notestine et al. 2013), we used the multi-channel dataset in a semi-automated workflow to measure total cerebral white matter and abnormal white matter (e.g., hyperintense regions on T2-weighted images) and subcortical (including caudate nucleus, putamen, nucleus accumbens, thalamus, and hypothalamic areas) and cerebral cortical gray matter volumes, as well as supratentorial cranial vault volumes to account for individual differences in head size. The workflow includes image inspection for motion and other artifacts, re-slicing to a standard space, intra-subject mutual information (MI) registration, bias correction with non-parametric, non-uniformity normalization (N3), removal of non-brain tissue, three-tissue segmentation (gray matter, white matter, and CSF), aWM designation, and anatomical labeling performed by trained anatomists. This approach includes the identification of regions of white matter with abnormal MR signal characteristics; these regions segmented as gray matter, but are anatomically located within the white matter.

#### Single-voxel MR spectroscopy

The details related to image sequences and analyses for single-voxel MRS are available elsewhere (Anderson et al. 2015); we provide an overview here. All imaging was performed using a standardized point-resolved (PRESS) protocol. Using LC Model with water scaling (using the unsuppressed water reference signal) allowed the quantification of absolute metabolite concentration (Provencher 2001). These metabolites included NAA, Cho, MI, and Cr. Their concentration was measured in three regions: mid-FGM, right FWM, and right BG. Due to demonstrated associations between Cr and HIV-related factors, such as plasma HIV RNA levels (Anderson et al. 2015), we used absolute values rather than ratios to Cr in our analyses. Water scaling allows for the examination of absolute metabolites. Only metabolite estimates for appropriately placed voxels with adequate spectra (standard deviation < 21) were used; therefore, sample size varied by MRS region or metabolite.

#### Statistical analysis

We explored three different models for MRI and MRS data. To address our primary aim, we used logistic regression with binary outcomes (NCI vs. NU). Included covariates were Nadir CD4 T cell count, plasma HIV RNA level, age, and scanner. To account for legacy effect, in particular, severity of immunosuppression prior to ART availability, we used CD4 nadir as a surrogate marker, and adjusted for it in all statistical analysis. To account for individual differences in head size, supratentorial vault size was included as a covariate in all statistical modeling of structural data. To obtain a normal distribution, in all analyses, HIV RNA level and structural MRI volumes were log transformed, and Nadir CD4 cell count was expressed as a square root. In regard to MRS data, to control for partial volumes, the proportion of relevant tissue volume within each voxel (e.g., amount of gray matter in FGM) was assessed by co-registering the MRS voxels to the morphometry volumes and included as a covariate in all statistical modeling. To address our secondary aim, we used a multinomial logistic regression with the neurocognitive impairment status which included NU, ANI, MND, and HAD categories. The covariates that were included are the same as the logistic regression stated above. Finally, we conducted a sub-analysis including only those with undetectable plasma HIV RNA; with the exception of HIV RNA level, all other covariates were included in these models.

## Results

### Study population

Demographics and clinical characteristics are summarized in Table 1. Participants were mostly men (81%) and had a mean age of 44.13 years. Forty percent met criteria for HAND, including 54 ANI, 37 MND, and 10 HAD. CSF viral escape was present in one participant and CSF-plasma discordance in four.

**Table 1:** Demographic and clinical characteristics. Values are mean (standard deviation), frequency count (percent), or median (IQR)

Full sample (*N* = 253)			Undetectable viral load subset (*n* = 126)
Age, M (SD)	44.1 (7.8)		45.1 (7.4)
Years of education, M (SD)	13.1 (2.4)		13.3 (2.3)
Ethnicity	African American	113 (44.7%)	52 (41.3%)
	Latino	28 (11.1%)	12 (9.5%)
	Other	5 (2%)	1 (0.8%)
	Non-Latino White	107 (42.3%)	61 (48.4%)
Gender	Men	205 (81%)	101 (80.2%)
	Women	48 (19%)	25 (19.8%)
AIDS status	AIDS	170 (67.2%)	92 (73.0%)
	Non-AIDS	83 (32.8%)	34 (27.0%)
HAND diagnosis	ANI	54 (21.3%)	35 (22.2%)
	MND	37 (14.6%)	25 (15.8%)
	HAD	10 (4%)	6 (3.8%)
	NU	152 (60.1%)	92 (58.2%)
cART history	Current	193 (76.3%)	123 (97.6%)
	Past	33 (13%)	2 (1.6%)
	Never	27 (10.7%)	1 (0.8%)
Nadir CD4, median (IQR)	150 (28, 284)		111 (15, 236)
Current CD4, median (IQR)	458 (289, 622)		518 (360, 684)
Detectable CSF viral load	67 (26.5%)		1 (0.8%)

### Structural MRI findings

**Fig. 1:**
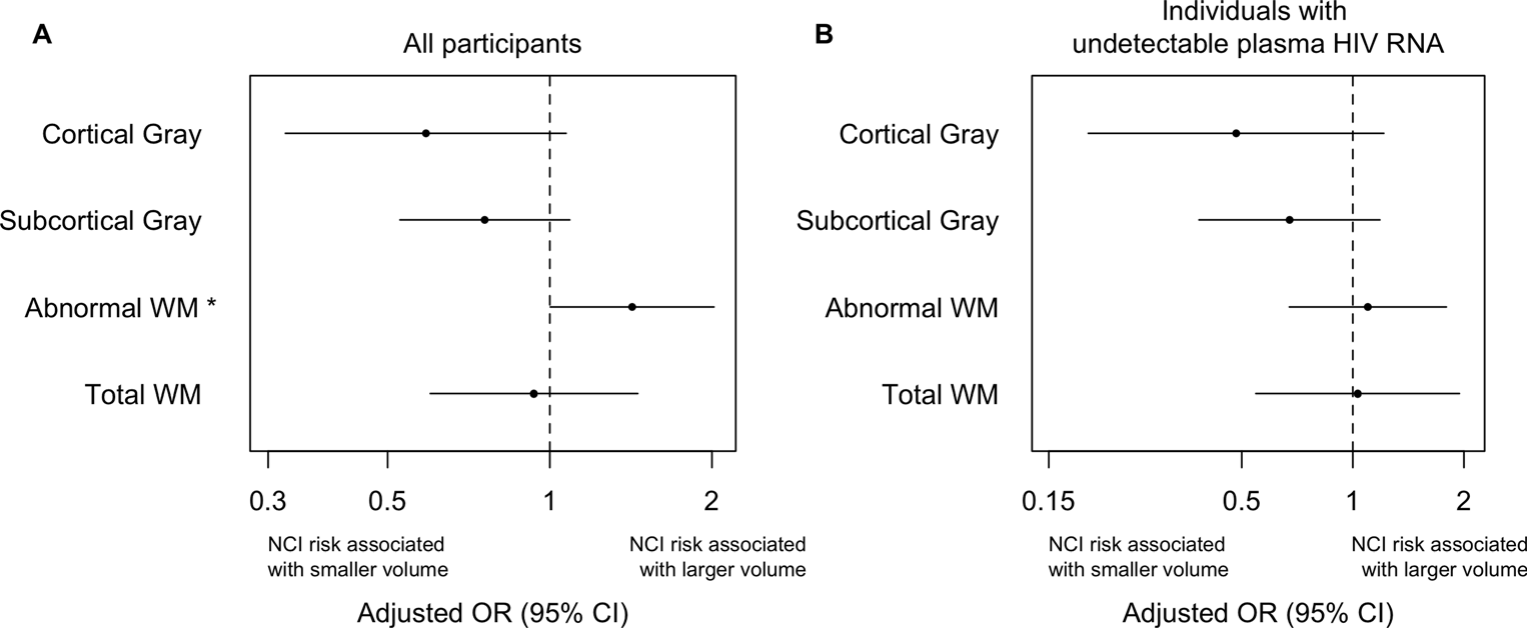
Structural MRI alterations associated with neurocognitive impairment (NCI = all HAND categories). **a** In all participants and **b** only in individuals with undetectable plasma HIV RNA. Adjusted binary logistic regression models were used to estimate the odds (95% CI) of HAND for each 1 standard deviation (SD) increase in log-transformed values of structural MRI volumes (mm^3^). Values less than 1.0 indicate that HAND was associated with smaller structural volumes, while values greater than 1.0 indicate that HAND was associated with larger volumes. *p < .05

**Fig. 2:**
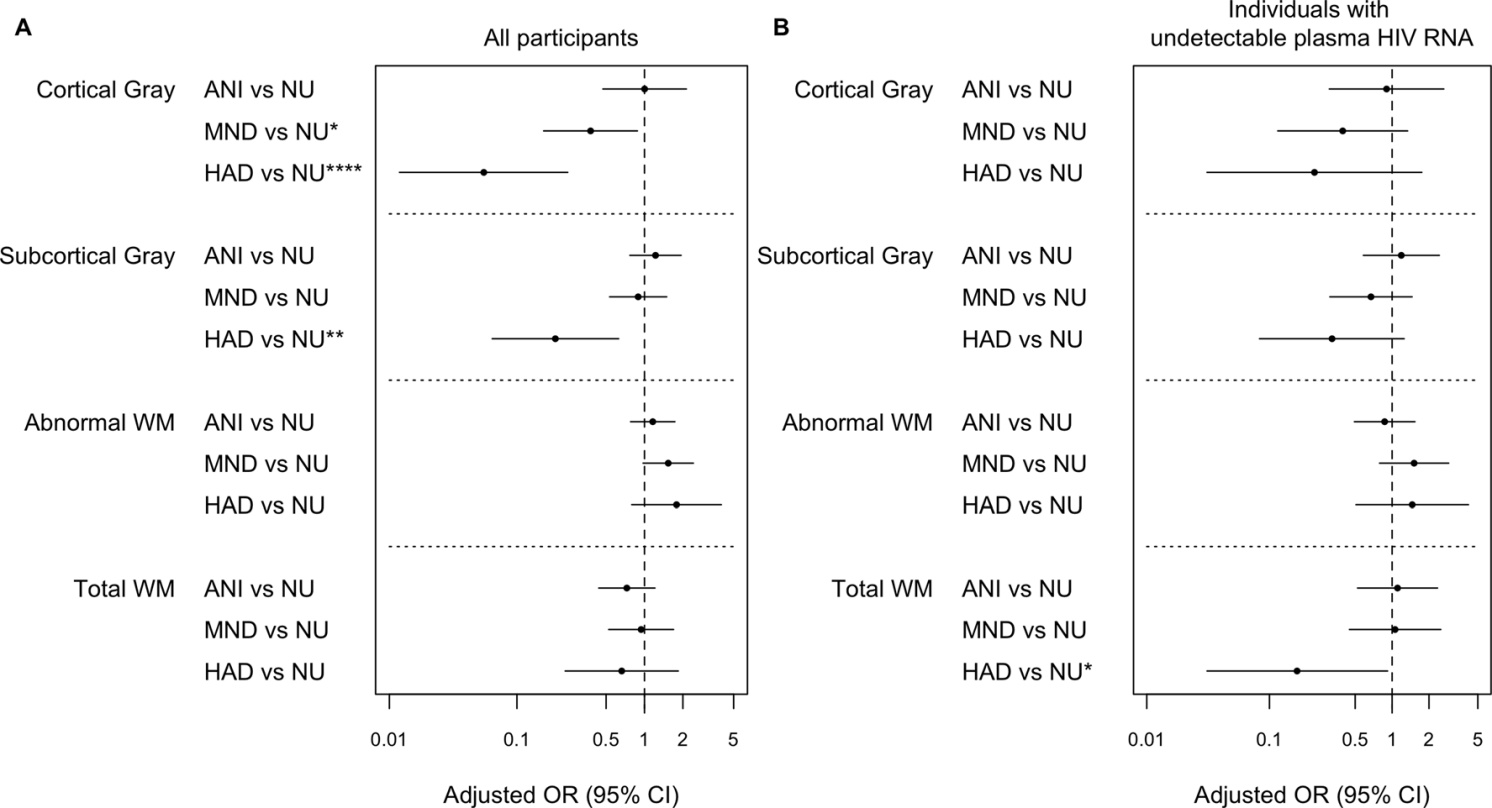
Structural MRI alterations associated with HAND classifications. **a** In all participants and **b** only in individuals with undetectable plasma HIV RNA. Adjusted multinomial logistic regression models predicting whether individuals were NU, ANI, MND, or HAD. The odds ratio and 95% CI for the effect of the measure is reported. The OR represents effect size per 1 SD increase in log-transformed values of structural MRI. ****p < .001; ***p < .005; **p < .01; *p < .05

**Fig. 3:**
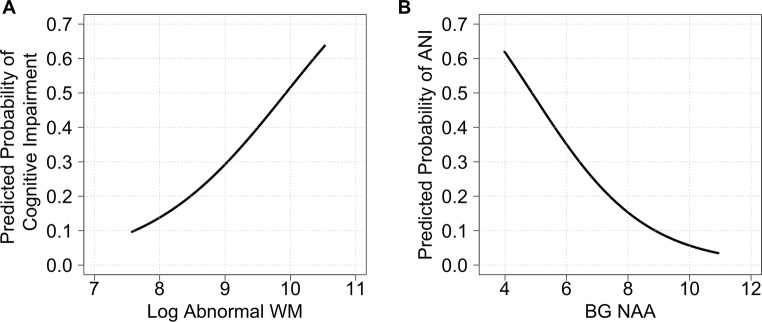
Probability of cognitive impairment (any HAND) plotted against amount of **a** abnormal white matter (log_10_ mm^3^) and **b** BG-NAA (unit-less absolute values derived using water scaling), after adjusting for nadir CD4 cell count, HIV RNA level count, age, scanner and supratentorial cranial vault size in SMRI and proportion of relevant tissue within each voxel in MRS. The probability curves shown were obtained by setting the covariates to their average values. *p* values for both relationships were significant (see text)

For all participants, the amount of aWM significantly differentiated NCI (all HAND subtypes together) from NU (*p* < .05; Fig. 1a). Increase of 1 log10 in aWM was associated with a higher likelihood (OR 2.6; 95% CI 1.01–6.6) of being cognitively impaired (Fig. 3a). We performed a multinomial regression analysis to examine differences between the three cognitive impairment HAND groups and NU (Fig. 2). No significant differences between ANI and NU were found in any structural MRI volume (Fig. 2a). However, HAD compared to the NU and ANI groups had smaller cortical and subcortical gray volumes. For every one standard deviation reduction in log-cortical gray volume, the odds of having HAD increased by 18 times. Finally, smaller cortical gray volumes were associated with higher odds of having MND as compared to NU (*p* < .05).

In analyses of individuals with undetectable plasma HIV RNA viral load, aWM volume no longer significantly differentiated NCI and NU (Fig. 1b), and NU and HAD only differed on total white matter volume (Fig. 2b).

### MRS findings

**Fig. 4:**
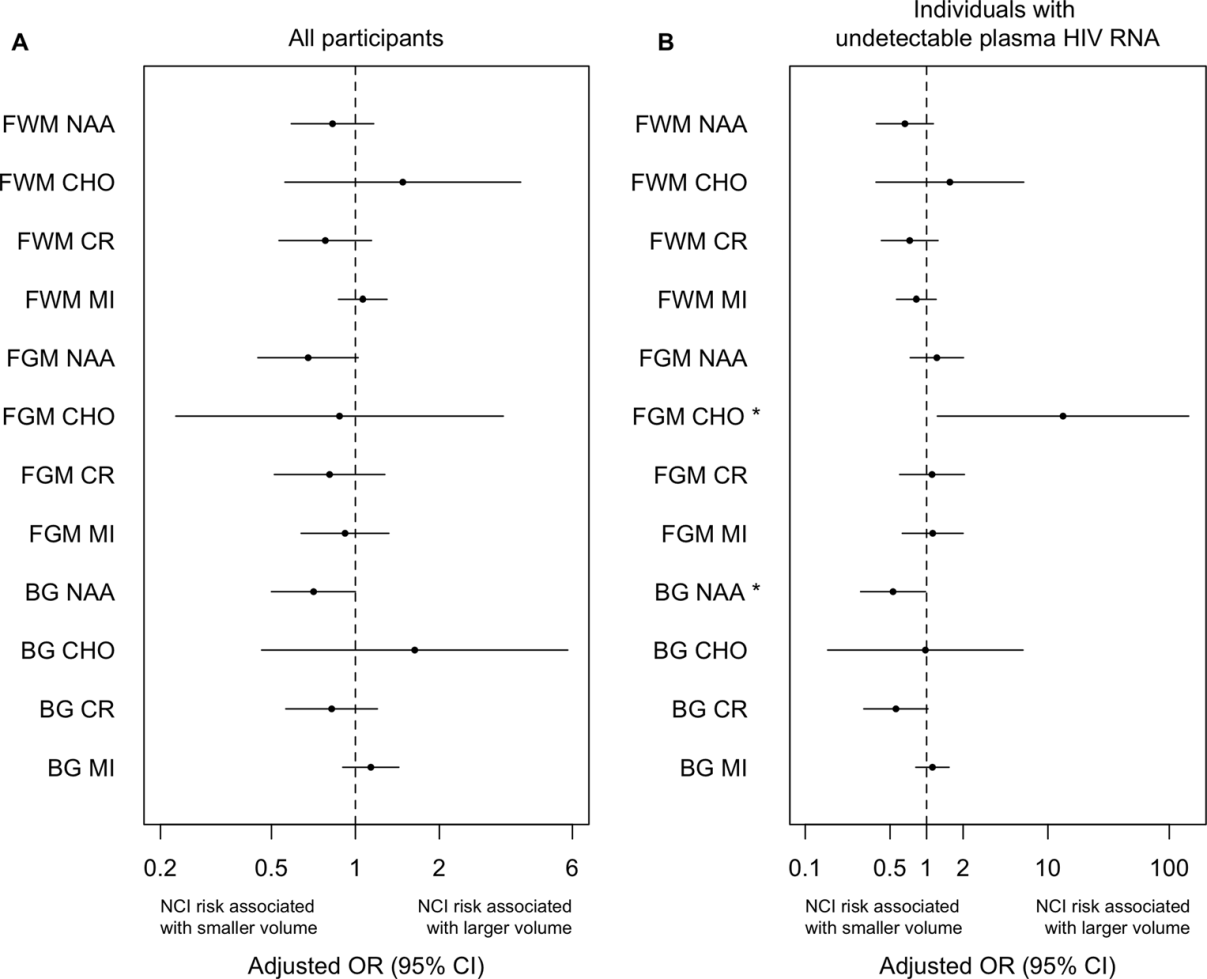
MRS alterations associated with neurocognitive impairment (NCI = all HAND categories). **a** In all participant and **b** in individuals with undetectable plasma HIV RNA. Adjusted binary logistic regression models predicting whether individuals were impaired or not. The odds ratio and 95% CI for the effect of the MRS measure is reported. *p < .05

**Fig. 5:**
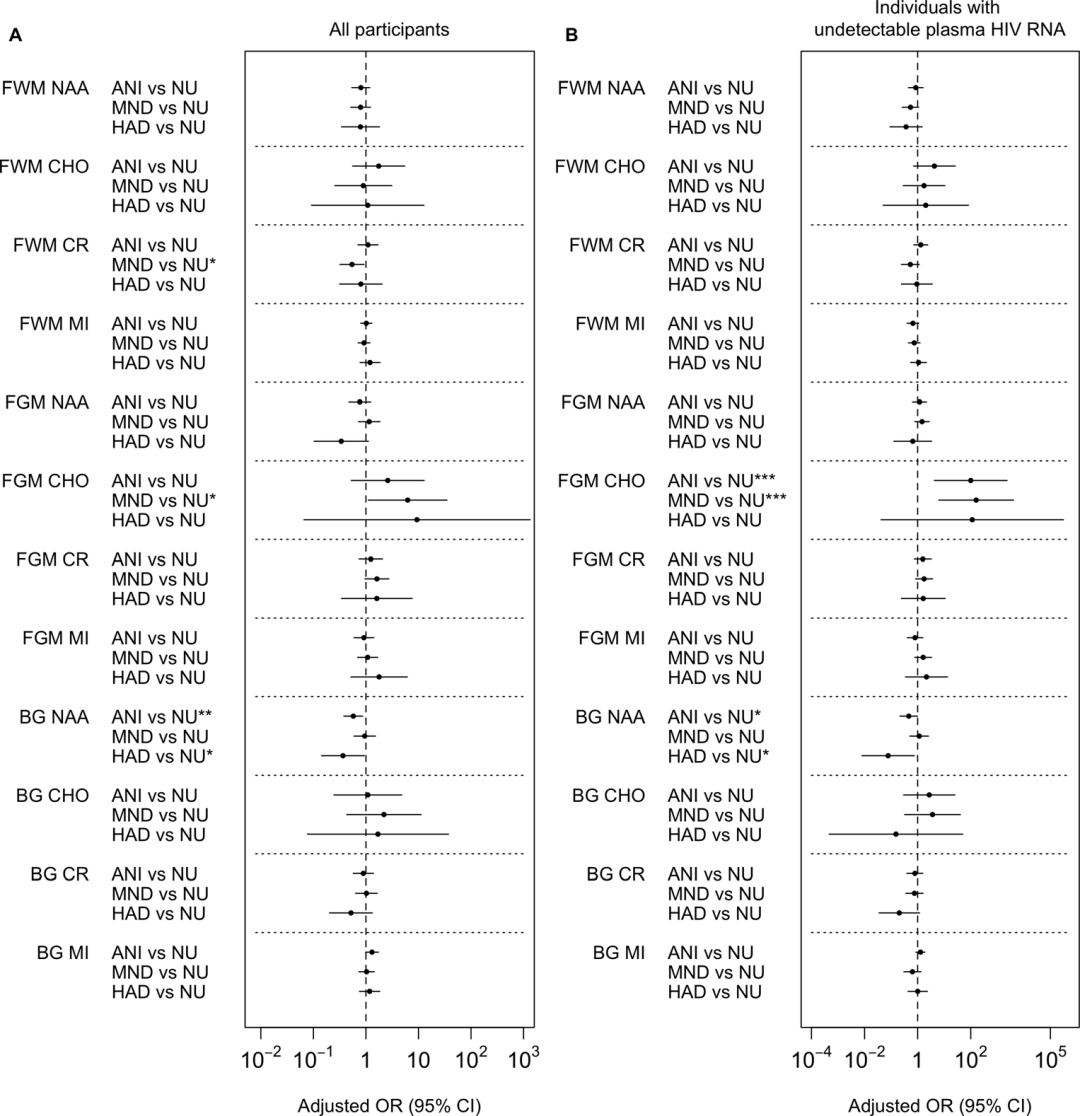
MRS alterations associated with HAND classifications. **a** In all participant and **b** only in individuals with undetectable plasma HIV RNA. Adjusted multinomial logistic regression models predicting whether individuals were NU, ANI, MND, or HAD. The odds ratio and 95% CI for the effect of the measure is reported. ****p < .001; ***p < .005; **p < .01; *p < .05

For all participants, NCI overall did not differ from NU in any MRS measure (Fig. 4a). In multinomial regression analysis of differences between the HAND groups, higher BG-NAA was associated with a lower likelihood of both ANI and HAD compared to NU (OR 0.58; 95% CI 0.38–0.87; OR 0.37; 95% CI 0.14–0.93, respectively; Fig. 5a). Lower BG-NAA was associated with a higher likelihood of having ANI (Fig. 3b). FWM-Cr was significantly lower in MND than NU with odds ratios of 0.54 (CI 0.32–0. 92) (Fig. 5a). FGM-Cho was higher in MND than in NU such that with every one-unit increase in FGM-Cho, the odds of having MND increased by 6.24 (CI 1.11–34.9; Fig. 5a).

In analyses of persons with undetectable plasma HIV RNA viral load, FGM-Cho was higher and BG-NAA lower in NCI relative to NU (Fig. 4b), unlike when all participants were included. The differences in BG-NAA between NU and both ANI and HAD persisted (*p* < .05) (Fig. 5b). FGM-Cho was significantly higher and BG-NAA was lower in NCI than in NU (*p* < .05). In addition, FGM-Cho was also significantly higher in both MND and ANI relative to NU (*p* < .005; Fig. 5b). No other effects were significant.

## Discussion

In this large, geographically diverse US sample of HIV+ persons, we found evidence for alterations in brain volumes and chemistry associated with neurocognitive impairment and specific HAND subtypes, even during successful viral suppression. As in other cART-era cohorts, the milder subtypes of HAND—ANI and MND—were much more frequent than HAD, but we observed MR differences in all forms of HAND. Structurally, HAD was distinguished from the less severe forms of HAND by greater loss of cortical and subcortical gray matter. HAND groups had more aWM than NU. Chemically, HAD and ANI had a lower marker of neuronal integrity (lower BG-NAA) compared to NU. These associations were not attributable to current virologic failure, current or past immunosuppression, or other relevant variables.

We found more abnormal, T2-hyperintense cerebral white matter (aWM) in HAND. The volume measurements used in our study were based on semi-automated, quantitative, multi-channel morphometry. By incorporating information from multiple sequences, our method measures additional WM alterations not observed on clinical MRI readings (Su et al. 2016; Fazekas et al. 1987); the latter are frequently referred to as white matter hyperintensities (WMHs). Other investigations have reported neurocognitive impairment to be associated with increased amounts of WMH in HIV-infected men (Su et al. 2016), and in older, virologically suppressed men (Watson et al. 2017). These two studies did not distinguish between HAND subtypes and did not systematically assess or exclude individuals with confounding neurocognitive comorbidities such as developmental learning disabilities and head injury (Antinori et al. 2007). Previous studies have identified several risk factors for higher volumes of aWM including lower nadir CD4 (Jernigan et al. 2011); we adjusted for nadir CD4 in our analyses. Related studies using diffusion tensor imaging to delineate white matter abnormalities have also shown these to be correlated with neurocognitive impairment in virally suppressed individuals (Cysique et al. 2017a).

The etiology of white matter abnormalities in HIV infection is uncertain. Proposed pathophysiologies include demyelination, inflammation, synaptodendritic injury, and microvascular alterations associated with concomitant cardiovascular risk factors, especially hypertension. cART also may contribute to synaptic injury via oxidative stress as has been demonstrated in vitro and in animal models (Akay et al. 2014). We previously reported that more aWM correlated with reduced dendritic density by MAP-2 immunostaining and with higher burden of HIV protein by gp41 immunoreactivity (Archibald et al. 2004). More WMHs are correlated with lower fractional anisotropy (FA) values on diffusion imaging, consistent with disrupted organization of white matter tracts (Kochunov et al. 2007). Indeed, a recent study found that lower FA in the cingulum and fornix correlated with cognitive impairment in virally suppressed HIV+ individuals (Cysique et al. 2017b). Finally, we previously reported that greater CD4 recovery during cART was associated with increases in aWM, suggesting that immune reconstitution may contribute to these lesions (Fennema-Notestine et al. 2013). The aWM observed here might reflect combinations of all of these alterations.

In contrast to a recent report (Kugathasan et al. 2017) in which 15% of patients showed CSF HIV escape or discordance, we found very few such instances (1.9%), and no evidence of an association with aWM. Several methodological differences may explain these discrepant findings. While the participants in the Kugathasan et al. study (Kugathasan et al. 2017) underwent MR evaluation after presenting with acute, subacute, or chronic neurological symptoms, we performed study protocol-determined MRs on ambulatory research study volunteers without reference to any specific neurological symptoms. Whereas Kugathasan et al. graded the presence or absence of diffuse white matter signal abnormalities and focal white matter lesions based on visual inspection by a neuroradiologist, we instead used a semi-automated, multi-channel volumetric assessment to quantify the total volume of abnormal white matter, defined by T1, proton density, and T2 characteristics.

Using MRS, we found lower NAA, a marker of worse neuronal integrity, in the BG of ANI and HAD compared to NU. This was true for both the entire study sample and the suppressed subset. These findings are similar to prior studies showing lower NAA most in subcortical brain regions in HIV+ individuals with poorer neurocognitive performance (Paul et al. 2008), mild cognitive difficulties (Chang et al. 2004), and HAD (Yiannoutsos et al. 2004). However, the prior studies did not employ the current HAND classification system, but rather used systems such as the older Memorial Sloan Kettering (MSK) AIDS Dementia Complex (ADC) scale (Mohamed et al. 2010). One study (Harezlak et al. 2011) found that patients with ADC had significantly lower BG-NAA/CR relative to HIV-uninfected controls and another found that individuals with HAD had lower NAA in the BG compared to individuals with mild cognitive impairment and those who were unimpaired (Mohamed et al. 2010). Finally, a recent longitudinal study of HIV+ individuals on stable cART showed that neurocognitive decline was associated with marginally significant longitudinal decreases in BG-NAA (Gongvatana et al. 2013) and another recent longitudinal study found sharply decreasing NAA in the posterior cingulate cortex in progressing HAND, relative to an HIV− group (Cysique et al. 2018).

In addition, we found significantly higher Cho, which may reflect neuroinflammation, in the frontal gray matter of the ANI and MND groups compared to NU. This was true for the subset with virologic suppression on cART; however, only the MND vs. NU comparison was significant in the overall study sample, a difference possibly attributable to greater variability in the unsuppressed subjects. Similarly, previous studies found increased Cho in frontal white matter in HIV+ individuals with poorer neurocognitive performance (Paul et al. 2008), mild cognitive difficulties (Chang et al. 2004), and HAD (Yiannoutsos et al. 2004). While we found significantly lower creatine in frontal white matter in MND compared to NU; this finding did not hold in the virally suppressed group; a recent longitudinal study of virally suppressed HIV+ individuals found more robust creatine differences with stable HAND showing decreasing creatine and incident HAND showing steep creatine reductions in frontal white matter relative to HIV− controls (Cysique et al. 2018).

Strengths of this study include a large, geographically and demographically diverse HIV+ cohort from which individuals with severe neurocognitive confounds were excluded. Whereas many previous studies used metabolite ratios (e.g., NAA/Cr), we used absolute metabolite quantitation, which has the advantages of smaller coefficients of variation, reduced bias when creatine concentrations are altered, and more reliable interpretation of spectral data (Minati et al. 2010; Jansen et al. 2006). Our study was limited by a lack of HIV-uninfected controls; therefore, we cannot reliably distinguish whether the neuroimaging correlates of neurocognitive impairment that we observed were related to HIV itself or to other factors present in the cohort. Prior studies have demonstrated structural MRI differences in HIV-infected individuals relative to seronegative controls in the post-cART era. These included atrophy in cortical and subcortical gray and white matter even in virologically suppressed cohorts (Ances et al. 2012; Cardenas et al. 2009; Becker et al. 2011). However, these studies did not evaluate whether these differences were linked to cognitive impairment. Another limitation of our study is its cross-sectional design, which precludes determination of when cerebral alterations may have occurred—for example, prior to or during cART treatment. Lastly, we did not control for multiple comparisons, which may increase the chance of type 1 error; however, the directions of the associations observed were as hypothesized (for example, increased FGM-Cho in MND and ANI compared to NU); in contrast, type 1 errors would be expected to yield random directionality.

In this diverse cohort of HIV+ individuals, we found MR evidence of brain alterations associated with neurocognitive impairment generally and with specific HAND subtypes, even during successful viral suppression. HAND was associated with more abnormal WM, increased MRS markers of neuroinflammation, and reduced markers of neuronal integrity. These different metabolic and structural markers may reflect processes that vary over time and across individuals. For example, structural changes like volume loss and white matter abnormalities might be preceded by increases in metabolic markers of inflammation. Overall, MR alterations linked to cognitive impairment in the cART era reflect inflammation, white matter disruption, and neuronal injury. These effects may result from one or a combination of the following: irreversible injury due to a prior history of untreated HIV infection, continuing accumulation of CNS injury even during treated HIV, HIV-associated CNS coinfections, or toxic effects of specific antiretroviral medications.

### Acknowledgments and support

This work was supported in part by awards from the National Institutes of Health for the CNS HIV Anti-Retroviral Therapy Effects Research (CHARTER) [N01 MH2205 and HHSN271201000036C to I. Grant], R01 MH107345 (Heaton, R.K./Letendre, S.L.), and P30 MH62512 (R. Heaton). The views expressed in this article are those of the authors and do not reflect the official policy or position of the US Government. The CNS HIV Anti-Retroviral Therapy Effects Research (CHARTER) group is affiliated with Johns Hopkins University; the Icahn School of Medicine at Mount Sinai; University of California, San Diego; University of Texas, Galveston; University of Washington, Seattle; and Washington University, St. Louis, and is headquartered at the University of California, San Diego, and includes the following: Director—Igor Grant, M.D.; Co-Directors—Scott L. Letendre, M.D., Ronald J. Ellis, M.D., Ph.D., Thomas D. Marcotte, Ph.D.; Center Manager—Donald Franklin, Jr.; Neuromedical Component—Ronald J. Ellis, M.D., Ph.D. (P.I.), J. Allen McCutchan, M.D.; Laboratory and Virology Component—Scott Letendre, M.D. (Co-P.I.), Sara Gianella Weibel, M.D. (Co-P.I.).; Neurobehavioral Component—Robert K. Heaton, Ph.D. (P.I.), J. Hampton Atkinson, M.D., Matthew Dawson; Imaging Component—Christine Fennema-Notestine, Ph.D. (P.I.), Rebecca Theilmann, Ph.D.; Data Management Component—Ian Abramson, Ph.D. (P.I.), Clint Cushman; Statistics Component—Florin Vaida, Ph.D. (P.I.), Ian Abramson, Ph.D.; Johns Hopkins University Site—Ned Sacktor (P.I.), Vincent Rogalski; Icahn School of Medicine at Mount Sinai Site—Susan Morgello, M.D. (Co-P.I.) and David Simpson, M.D. (Co-P.I.), Letty Mintz, N.P.; University of California, San Diego Site—J. Allen McCutchan, M.D. (P.I.); University of Washington, Seattle Site—Ann Collier, M.D. (Co-P.I.) and Christina Marra, M.D. (Co-P.I.), Sher Storey, PA-C.; University of Texas, Galveston Site—Benjamin Gelman, M.D., Ph.D. (P.I.), Eleanor Head, R.N., B.S.N.; and Washington University, St. Louis Site—David Clifford, M.D. (P.I.), Mengesha Teshome, M.D.

## Abbreviations

HAND: HIV-Associated neurocognitive disorder
NCI: Neurocognitive impairment
NU: Neurocognitively unimpaired
ANI: Asymptomatic neurocognitive disorder
MND: Mild neurocognitive disorder
HAD: HIV-associated dementia
cART: Combined antiretroviral therapy
MRS: Magnetic resonance spectroscopy
aWM: Abnormal white matter
CSF: Cerebrospinal fluid
NAA: *N*­Acetyl aspartate
Cho: Choline
MI: Myoinositol
Cr: Creatine
FGM: Frontal gray matter
FWM: Frontal white matter
BG: Basal ganglia
